# VBP1 promotes tumor proliferation as a part of the hypoxia-related signature in esophageal squamous cell carcinoma

**DOI:** 10.1007/s13577-024-01068-9

**Published:** 2024-05-03

**Authors:** Huikai Miao, Wuyou Gao, Leqi Zhong, Hongmu Li, Dongni Chen, Chunmei Xu, Zhesheng Wen, Youfang Chen

**Affiliations:** 1 Department of Thoracic Surgery, Shandong Provincial Hospital affiliated to Shandong First Medical University, Jinan, China; 2 Department of Thoracic Oncology, State Key Laboratory of Oncology in South China, Collaborative Innovation Center for Cancer Medicine, Sun Yat-Sen University Cancer Center, 651 Dongfeng East Road, Guangzhou, China; 3 Department of Thoracic Surgery, Nanfang Hospital, Southern Medical University, Guangzhou, China; 4 Department of Endocrinology, Shandong Provincial Hospital affiliated to, Shandong First Medical University, Jinan, China

**Keywords:** Esophageal squamous cell carcinoma, Hypoxia, Prognosis, VBP1, Proliferation

## Abstract

Esophageal squamous cell carcinoma (ESCC) is a common malignant tumor in East Asia. Hypoxia, a hallmark of solid tumors, significantly alters redox homeostasis inside tumor microenvironment. This alteration drives tumor proliferation, invasion, and metastasis, leading to poor prognostic outcomes. However, the role of hypoxia-related genes in ESCC remains poorly understood. We employed RNA sequencing to identify differentially expressed genes in ESCC. Clinical data, transcriptome profiles, and a hypoxia-related gene set were extracted from open-source databases. A prognostic model was constructed using least absolute shrinkage and selection operator (LASSO) regression, which was then validated through Cox regression analysis. Within this prognostic model, we pinpointed and investigated a key hypoxia-related gene affecting prognosis. The gene's expression was validated using real-time PCR and immunohistochemistry in both esophageal carcinoma and normal tissues. Tumor proliferation was examined through in vitro and in vivo assays, including the Cell Counting Kit-8, EdU, colony formation, and subcutaneous tumor models. A robust four-gene prognostic model (VBP1, BGN, CDKN1A, and PPFIA1) was successfully constructed and validated. Among these, VBP1 emerged as a key gene, exhibiting high expression levels that correlated with poor prognosis in ESCC. Functional experiments confirmed that VBP1 significantly accelerated tumor proliferation both in vitro and in vivo. VBP1 is identified as a pivotal gene within the hypoxia-related prognostic signature, and it significantly promotes tumor proliferation in ESCC.

**Supplementary Information:**

The online version contains supplementary material available at 10.1007/s13577-024-01068-9.

## Introduction

Esophageal carcinoma is one of the most common malignant tumors, ranking sixth in overall mortality rates [1]. The predominant pathological type in China is esophageal squamous cell carcinoma (ESCC) [2]. Traditional treatments such as surgery, radiotherapy, and chemotherapy often fall short due to the high incidence of recurrence and metastasis in ESCC patients [3]. Consequently, the 5-year overall survival rate does not exceed 15–20% [4].

Hypoxia, characterized by low oxygen tension in tissues, is a common feature of solid tumors, including ESCC [5]. This condition disrupts oxygen homeostasis, creating redox stress in the tumor microenvironment. Such altered redox conditions propel tumor progression and metastasis, which are associated with poor prognostic outcomes [6, 7]. Despite its clinical relevance, the molecular mechanisms underpinning this association remain elusive [8]. Meanwhile, the tumor-node-metastasis (TNM) stage system is the primary prognostic tool in clinical practice. However, this system is not universally applicable due to tumor heterogeneity [9].

In our study, we constructed a four-gene prognostic model that assesses clinicopathological features and predicts the overall survival of ESCC patients. Within this hypoxia-related gene model, VBP1 emerged as a pivotal gene that promotes tumor proliferation in ESCC.

VBP1 (Hippel-Lindau-binding protein 1, also known as prefoldin 3) serves as a subunit of the prefoldin complex [10]. It was initially identified as a cochaperone protein that binds to the C-terminal end of pVHL (von Hippel-Lindau tumor suppressor protein) [11]. pVHL functions as an E3-ubiquitin ligase, degrading HIF-1α (hypoxia-inducible factor 1 subunit alpha) in an oxygen-dependent manner. HIF-1α drives adaptive responses to oxidative stress through nuclear translocation and gene expression regulation [12]. Specifically, HIF-1α modulates glucose metabolism, angiogenesis, and tumor proliferation in hypoxic environments [13]. However, the role of VBP1, a hypoxia-related gene, remains unexplored in the context of ESCC.

## Materials and methods

### Identification of hypoxia-related differentially expressed genes (DEGs) between ESCC and normal tissues

RNA sequencing (RNA-Seq) was employed to identify DEGs between six pairs of ESCC and adjacent normal tissues [14]. RNA-Seq tissues were collected from patients who received esophagectomy surgery in our cancer center in 2019. RNA-Seq were conducted and analyzed by Genminix-GCBI (Shanghai, China) with a screening criteria of an absolute log2-fold change (FC) > 1 and an adjusted P-value < 0.05. A hypoxia-related genes set was retrieved and downloaded from Gene Set Enrichment Analysis (GSEA) database [15]. The intersection of the two gene sets yielded the hypoxia-related DEGs.

### Construction and validation of a prognostic signature

The mRNA expression profiles of ESCC patients and clinical data were retrieved from the Gene Expression Omnibus (GEO) and The Cancer Genome Atlas (TCGA) databases. Firstly, the univariate Cox regression analysis was conducted to identify the prognosis-related genes among the hypoxia-related DEGs using the “survival” R package on the GEO dataset. Subsequently, the Least absolute shrinkage and selection operator (LASSO) Cox regression was employed to narrow down the candidate genes via the “glmnet” R package [16]. The hypoxia-related prognostic gene signature was then determined based on the regression coefficient (β) and each gene's expression level, adhering to the proportional hazards assumption. The formula for risk score is articulated as: Risk score = β Gene1 × Expression level of gene1 + … + β Gene n × Expression level of gene n. This led to the categorization of patients into high-risk and low-risk groups, defined by the optimal cut-off value. The Kaplan–Meier survival analysis further assessed the predictive accuracy of this prognostic model in both the GEO and TCGA datasets.

Subsequently, we employed both univariate and multivariate Cox proportional hazard regression analyses to compare the prognostic gene signature's predictive capabilities against other clinical attributes. We analyzed the hazard ratio (HR) and 95% confidence intervals (CI) for ten primary clinical and prognostic variables, including age, sex, smoking habits, alcohol consumption, tumor location, grade, TNM stage, tumor stage, lymph node stage, and the hypoxia-related signature risk scores. Additionally, we visualized the risk using the “pheatmap” R package and evaluated the sensitivity and specificity of the gene signature with ROC curve analysis, facilitated by the “survival ROC” R package [17].

### Functional enrichment analysis

To understand the biological functions and pathways related to the hypoxia signature, we conducted gene set enrichment analysis (GSEA) on the high- and low-risk groups using GSEA software [15].

### Tumor specimens and cell culture

Tumor and adjacent normal tissues were collected from ESCC-diagnosed patients at our center. Patients were informed in alignment with the guidelines sanctioned by the Ethics Committee of Sun Yat-sen University Cancer Center. The corresponding clinical information includes age, gender, tumor grade, lymph node metastasis, AJCC TNM stages, and survival outcomes. Patients with insufficient clinical data were excluded. Propensity scores were estimated using age, gender (male versus female), TNM stage (I, II, III, IV), Tumor stage (T1, T2, T3, T4), Lymph Node stage (N0, N1, N2, N3), Metastasis stage (M0, M1) and treatment method (surgery, surgery + postoperative adjuvant chemotherapy). KYSE30 and KYSE150 were obtained from the Department of Experimental Research at the Sun Yat-sen University Cancer Center in Guangzhou, China. Cells were cultured in Dulbecco’s modified Eagle’s medium (DMEM) enriched with 10% FBS, and maintained at 37 °C in an atmosphere containing 5% CO_2_.

### Chemical and hypoxia treatment

A 25 mM stock solution of cobalt chloride (CoCl_2_, Sigma-Aldrich, Germany) was prepared using sterile distilled water (dH_2_O) and subsequently diluted in the medium to achieve desired concentrations. To simulate an anoxic environment, KYSE30 and KYSE150 cells underwent treatment with varying concentrations (0, 100, 200, 300, 400 umol/L) of CoCl_2_ for 24 h [18].

### Immunohistochemical assays

Immunohistochemical (IHC) analysis was performed according to the manufacturer’s instructions. Comprehensive details of antibodies and antigen retrieval techniques are available in Supplementary Table 1. For the survival analysis, ESCC patients from SYSUCC were grouped based on VBP1 expression levels, which was defined according to the following criteria: The intensity of VBP1 expression was scored as zero, negative; one point, weak staining; two points, mild staining; three points, strong staining. The positive stained area percentage (PSAP) of VBP1 expression was scored as 1, 0–25%; 2, 25%–50%; 3, 50%–75% and 4, 75%–100%. VBP1 IHC score = Intensity score × PSAP score. Patients were divided into high and low score groups by VBP1 IHC score and the Kaplan–Meier survival curves were analyzed using the log-rank test. The staining intensity was scored by two pathologists independently.

### Quantitative real-time PCR (qRT-PCR)

To assess the mRNA expression difference of VBP1 between ESCC and normal tissues, real-time RT-PCR was conducted according to the manufacturer’s guidelines. We extracted the total RNA from cells and tissues using the TRIzol reagent method. The cDNA was synthesized from 1 μg of cellular RNA. The RT-qPCR process was then executed on a Light-Cycler480II (Roche) using SYBR Green Master Mix and specific primers (details in Supplementary Table 2).

### Western blot

Following the manufacturer’s protocol, western blotting was carried out. In brief, cell lysates were prepared with RIPA Lysis Buffer (Beyotime, China) and protein quantification was done using the BCA Protein Kit (Beyotime, China). The cell lysates and immunocomplexes were then processed on SDS-PAGE and transferred to a PVDF membrane. These membranes, post-transfer, were blocked using QuickBlock™ Blocking Buffer (Beyotime, China), followed by an overnight incubation at 4 °C with specific antibodies. In the subsequent day, after incubation with secondary antibodies, immunoblots were visualized. The list of antibodies is provided in Supplementary Table 1.

### Small interfering RNAs (siRNAs) and gene knockdown

VBP1 and control siRNA were purchased from GenePharma (Suzhou, China). The VBP1 siRNA mediated gene knockdown in KYSE30 and KYSE150 cells was achieved with Lipofectamine®3000 (Invitrogen, USA), as described in Supplementary Table 3.

### Retroviruses and stable cell line

To establish KYSE30 cell lines that consistently overexpress VBP1, we utilized VBP1 recombinant retroviruses sourced from GenePharma (Suzhou, China). KYSE30 cells were infected with these retroviruses in the presence of polybrene. Following a 48-h infection period, we selected cells using 2 μg/mL puromycin to derive stable VBP1 over-expressing cell lines.

### Cell proliferation assays

The proliferation of KYSE30 and KYSE150 cells was measured using a Cell Counting Kit-8 kit (CCK-8, Yeasen, Shanghai, China). In the first day, 1000 cells per well were cultured in a 96-well plate with 100 μL DMEM medium. On subsequent days, we prepared a working solution by adding the CCK-8 reagent to the medium as instructed by the manufacturer. This solution was added to each well and incubated for 1.5 h, after which it was measured at 450 nm absorbance. CCK-8 assays were performed in three biological replicates.

### EdU assay

KYSE30 and KYSE150 cells were cultivated in 6-well plates and exposed to 2000 μL of medium containing 10 μM EdU, in line with the manufacturer’s instructions (BeyoClick™ EdU Cell Proliferation Kit with Alexa Fluor 594, Beyotime, China). After a 2-h incubation, cells were fixed and processed using Immunol Staining Fix Solution and Immunostaining Permeabilization Buffer, both from Beyotime, China. Cell nuclei were stained with Hoechst dye 33,342. Images were captured from three randomly chosen areas for each group, and the proliferation rate was calculated. EdU assays were performed in three biological replicates.

### Colony formation

KYSE30 and KYSE150 cells were cultured in 6-well plates. Following 14 days of the specified treatment, colonies were visualized using hematoxylin staining. This procedure was replicate in triplicate.

### Xenograft mouse model

For in vivo assessment, 4-week-old male nude mice (BALB/c-nu, Vital River Laboratory Animal Technology, Zhejiang, China) were utilized. KYSE30 cells (5 × 10^6^ cells per mouse) were injected subcutaneously in the right rear back region. Mice were then grouped into two categories: 8 for the control (NC) group and 8 for the VBP1 overexpression (OE) group. Both body weight and tumor sizes were observed every four days, with tumor volume calculated using the given formula, volume = 1/2 × length × (width)^2^. On the 18th day, tumors were extracted for further analysis using hematoxylin and eosin (HE) staining and immunohistochemistry.

### Statistical analysis

The SPSS 23.0 software program (IBM SPSS) and Graphpad Prism 7 (GraphPad Software Inc., CA, USA) were used for data analysis. Data are expressed as means ± standard deviation and P < 0.05 was considered statistically significant. Chi-squared or Fisher's exact tests were used to compare the categorical variables, and Student's t test was chosen to compare the difference in measurement data between two groups. The survival analyses were estimated by the Kaplan–Meier method, and the comparison was evaluated by the log-rank test. The univariate and multivariate analyses were conducted using a model of Cox's proportional hazards regression.

## Results

### Construction of a hypoxia‑related prognosis signature in ESCC

**Fig. 1:**
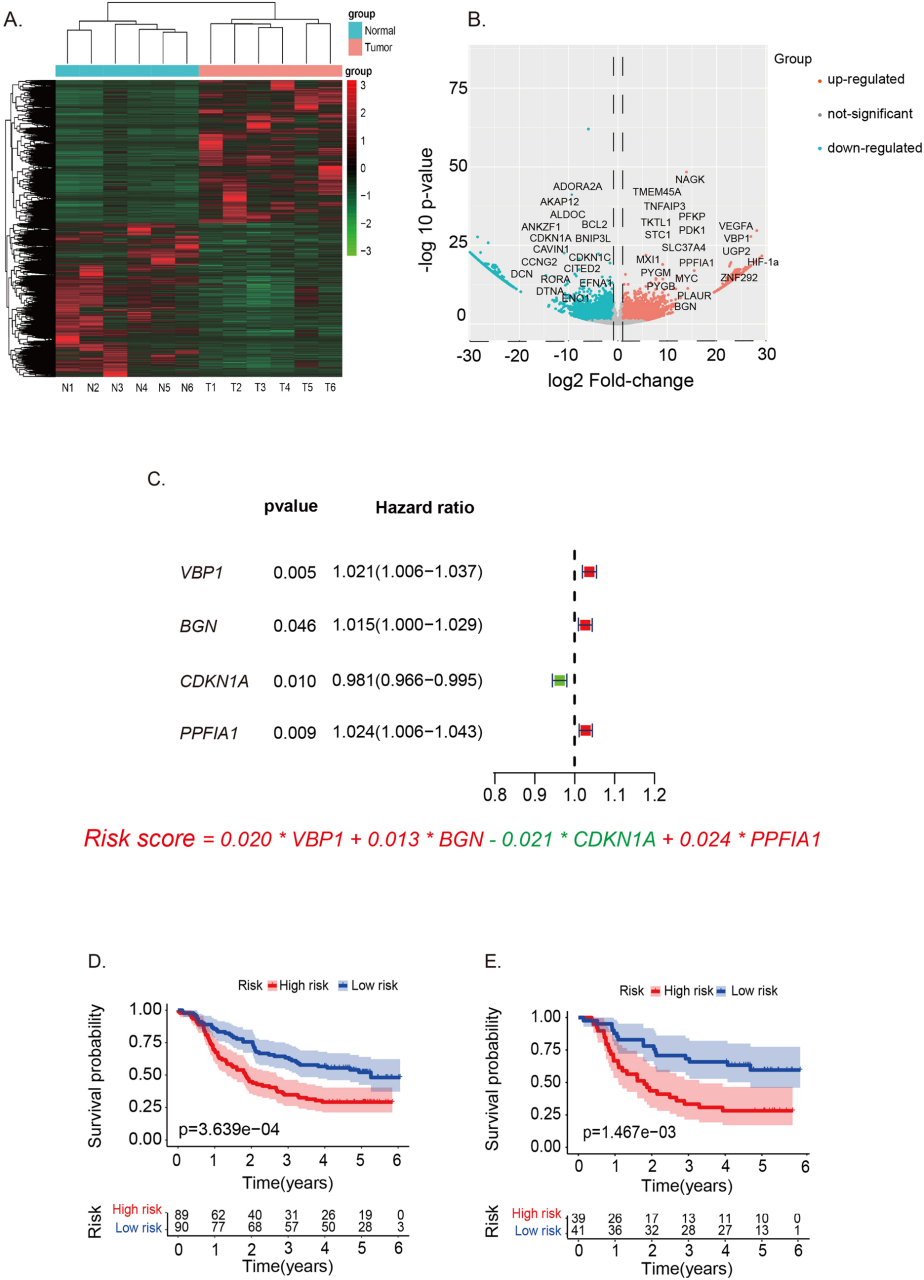
Construction of the hypoxia‑related prognosis signature in ESCC. **A** Heat map of DEGs in six pairs of ESCC and normal tissues. **B** Volcano plot of hypoxia-related DEGs in ESCC and normal tissues. **C** Forest plot for multiple Cox regression of the hypoxia-related signature. **D** Kaplan–Meier curve of the hypoxia-related signature in GEO ESCC patients (log rank test, p = 3.639e-04). **E** Kaplan–Meier curve of the hypoxia-related signature in TCGA ESCC patients (log rank test, p = 1.467e-03). *DEGs* differentially expressed genes, *ESCC* esophageal squamous cell carcinoma, *T* tumor, *N* normal tissue, *GEO* Gene Expression Omnibus database, *TCGA* The Cancer Genome Atlas database

In the study of ESCC, DEGs (|logFC|> 1, P < 0.05) were identified through RNA sequencing (RNA-Seq) from six pairs of ESCC and their adjacent noncancerous tissues (Fig. 1A). A subset of 61 DEGs, specifically linked to hypoxia in ESCC, was subsequently isolated (Fig. 1B).

Following univariate Cox regression and Lasso analysis, we shortlisted four genes (VBP1, BGN, CDKN1A, and PPFIA1) to construct a prognostic signature. This signature is articulated as: Risk Score = (0.020 × Expression level of VBP1) + (0.013 × Expression level of BGN) − (0.021 × Expression level of CDKN1A) + (0.024 × Expression level of PPFIA1) (Fig. 1C). Using this score, ESCC patients from the GEO (GSE53625) and TCGA databases were categorized into high- and low-risk groups based on the optimal cut-off value (Table 1). The Kaplan–Meier survival curves revealed a notably lower OS in the high-risk group compared to the low-risk group in the GEO dataset (P < 0.001, Fig. 1D). This prognostic signature’s predictive prowess was subsequently confirmed in the TCGA database (P < 0.01, Fig. 1E).

**Table 1:** Clinical characteristic of GEO, TCGA, and SYSUCC cohort

	GEO cohort	TCGA cohort	SYSUCC cohort
No. of patients	179	86	89
Age/years (median, range)	52 (36–82)	NA	65 (41–78)
Gender			
Female	33 (18.4%)	14 (16.3%)	25 (28.1%)
Male	146 (81.6%)	72 (83.7%)	64 (71.9%)
Tumor			
T 1–2	39 (21.8%)	38 (44.2%)	58 (65.2%)
T 3–4	140 (78.2%)	48 (55.8%)	31 (34.8%)
Node			
N 0–1	145 (81.0%)	77 (89.5%)	41 (46.1%)
N 2–3	34 (19.0%)	9 (10.5%)	48 (53.9%)
Metastasis			
M 0	179 (100%)	82 (95.3%)	89 (100%)
M 1	0	4 (4.7%)	0
TNM stage			
I	10 (5.6%)	6 (7.0%)	20 (22.5%)
II	77 (43.0%)	43 (50.0%)	44 (49.4%)
III	92 (51.4%)	63 (73.3%)	25 (28.1%)
IV	0	4 (4.7%)	0
Survival time/months(median, range)	32.5 (1.43–72.6)	13.0 (1.1–69.0)	16.4 (2.3–49.1)

*SYSUCC* Sun Yat-sen University Cancer Center, *NA* not available

### Evaluating the prognostic signature's independent contribution to OS prediction

**Fig. 2:**
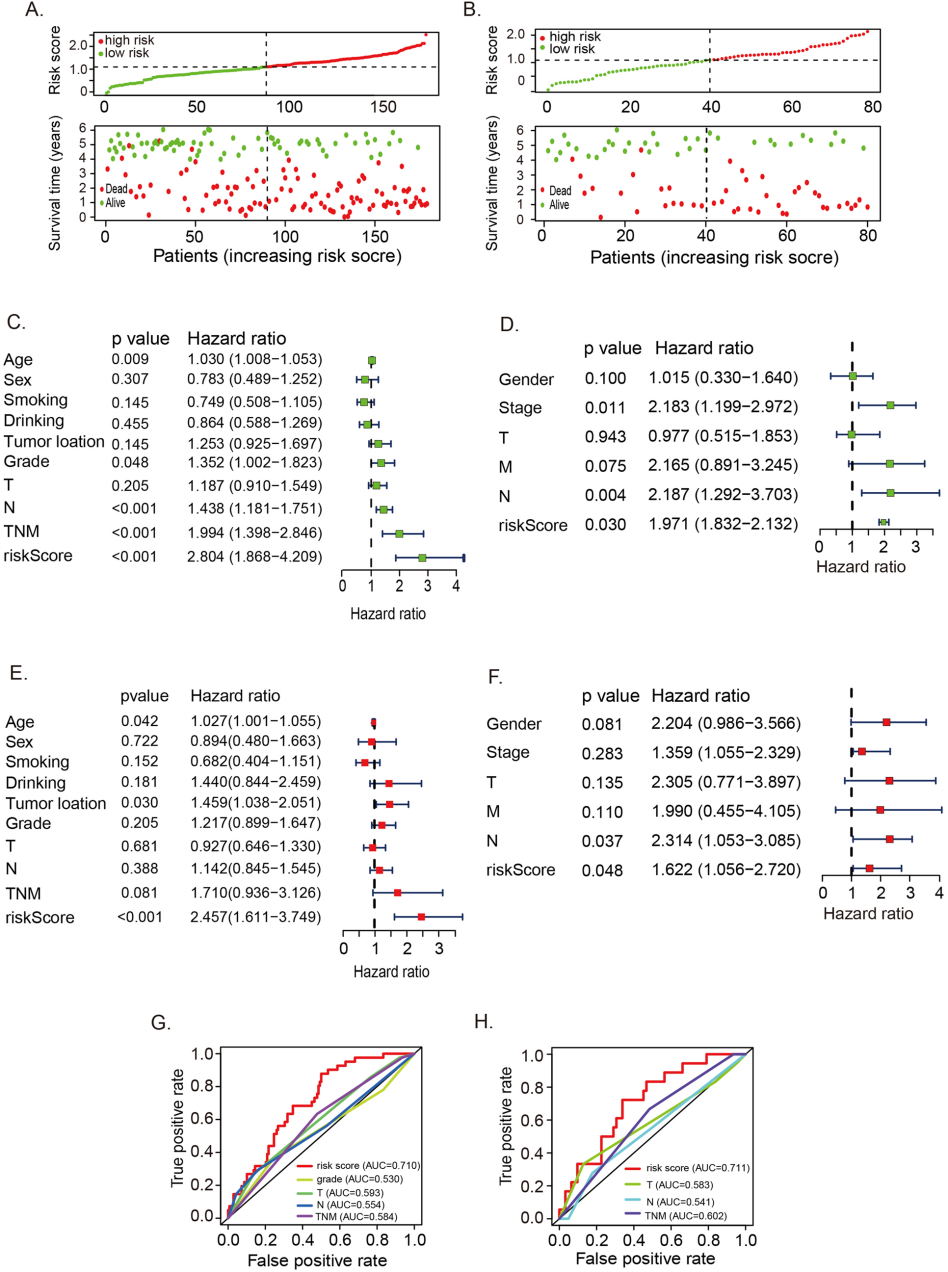
Evaluation of the hypoxia‑related prognosis signature in ESCC. **A** Risk-plot of the hypoxia-related signature in GEO ESCC patients. **B** Risk-plot of the hypoxia-related signature in TCGA ESCC patients. **C** Forest plot of univariate Cox regression of the hypoxia-related signature in GEO ESCC patients. **D** Forest plot of univariate Cox regression of the hypoxia-related signature in TCGA ESCC patients. **E** Forest plot of multivariate Cox regression of the hypoxia-related signature in GEO ESCC patients. **F** Forest plot of multivariate Cox regression of the hypoxia-related signature in TCGA ESCC patients. **G** ROC curve of the hypoxia-related signature in GEO ESCC patients (AUC = 0.7110). **H** ROC curve of the hypoxia-related signature in TCGA ESCC patients (AUC = 0.7111). *ROC* receiver operating characteristic, *AUC* area under the curve, *T* tumor, *N* lymph node

In the risk plots from both GEO (Fig. 2A) and TCGA (Fig. 2B) databases, there was an observable increase in patient mortality correlating with the risk score. Following both univariate (GEO in Fig. 2C, TCGA in Fig. 2D) and multivariate (GEO in Fig. 2E, TCGA in Fig. 2F) Cox regression analyses, the risk score emerged as an independent prognostic factor for OS when contrasted with other conventional clinical attributes. Notably, the AUCs of the four-gene prognostic model surpassed those of clinical characteristics, suggesting superior specificity and sensitivity (Fig. 2G, 2H).

**Fig. 3:**
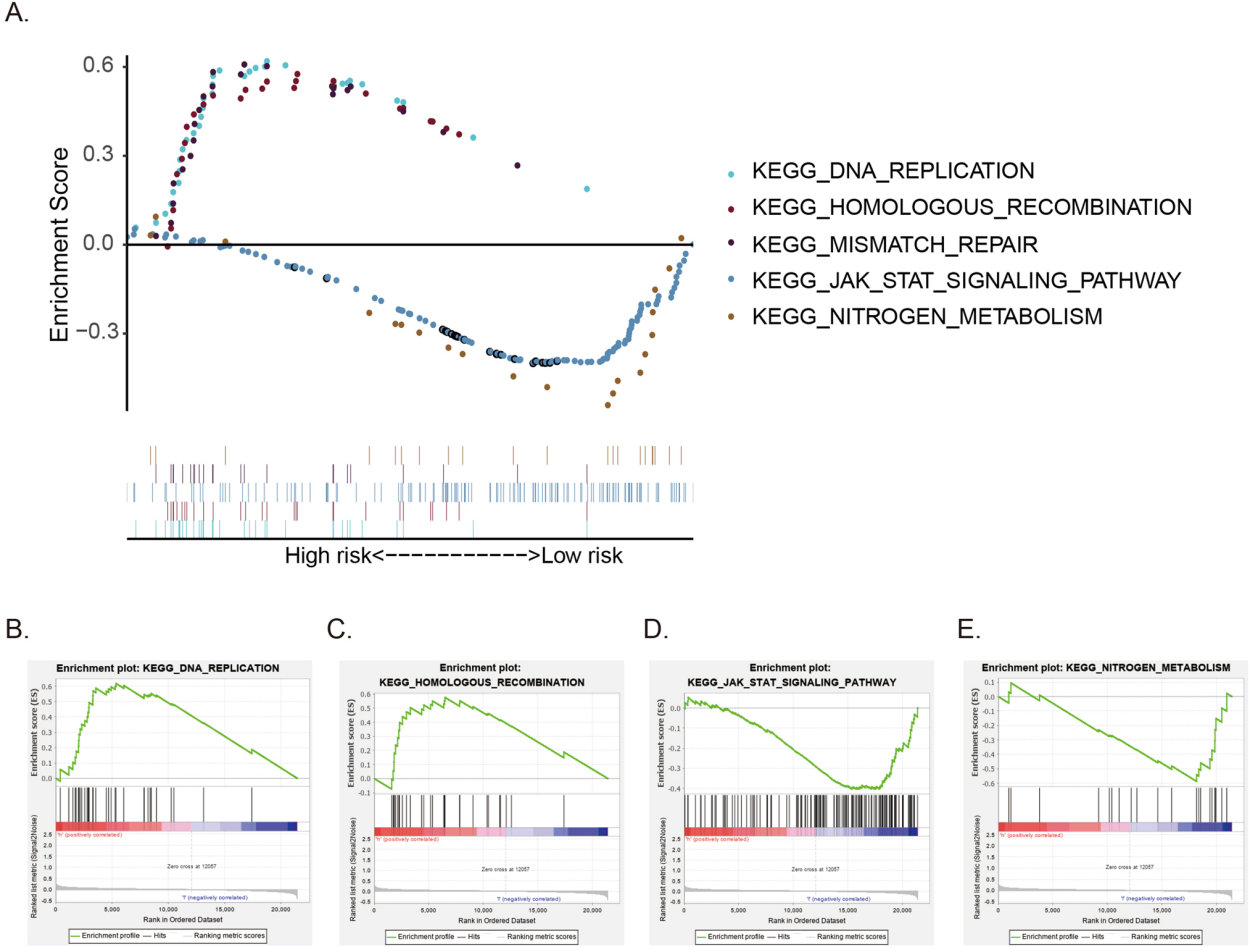
Gene set enrichment analysis of the hypoxia-related signature. **A** The multiple gene set enrichment analysis of the hypoxia-related signature. **B** The enrichment of DNA replication in the hypoxia-related signature high-risk group. **C** The enrichment of homologous recombination in the hypoxia-related signature high-risk group. **D** The enrichment of JAK-STAT signaling pathway in the hypoxia-related signature low-risk group. **E** The enrichment of nitrogen metabolism in the hypoxia-related signature low-risk group

To elucidate the biological functions of the four genes, we assessed the gene expression disparities between the low- and high-risk groups. Through the analysis of DEGs, GSEA revealed that DNA replication and homologous recombination pathways were notably overrepresented in high-risk patients. Conversely, the low-risk cases exhibited enrichments in the JAK-STAT signaling pathway and nitrogen metabolism (Fig. 3).

### VBP1 emerged as a pivotal gene within the prognostic signature

**Fig. 4:**
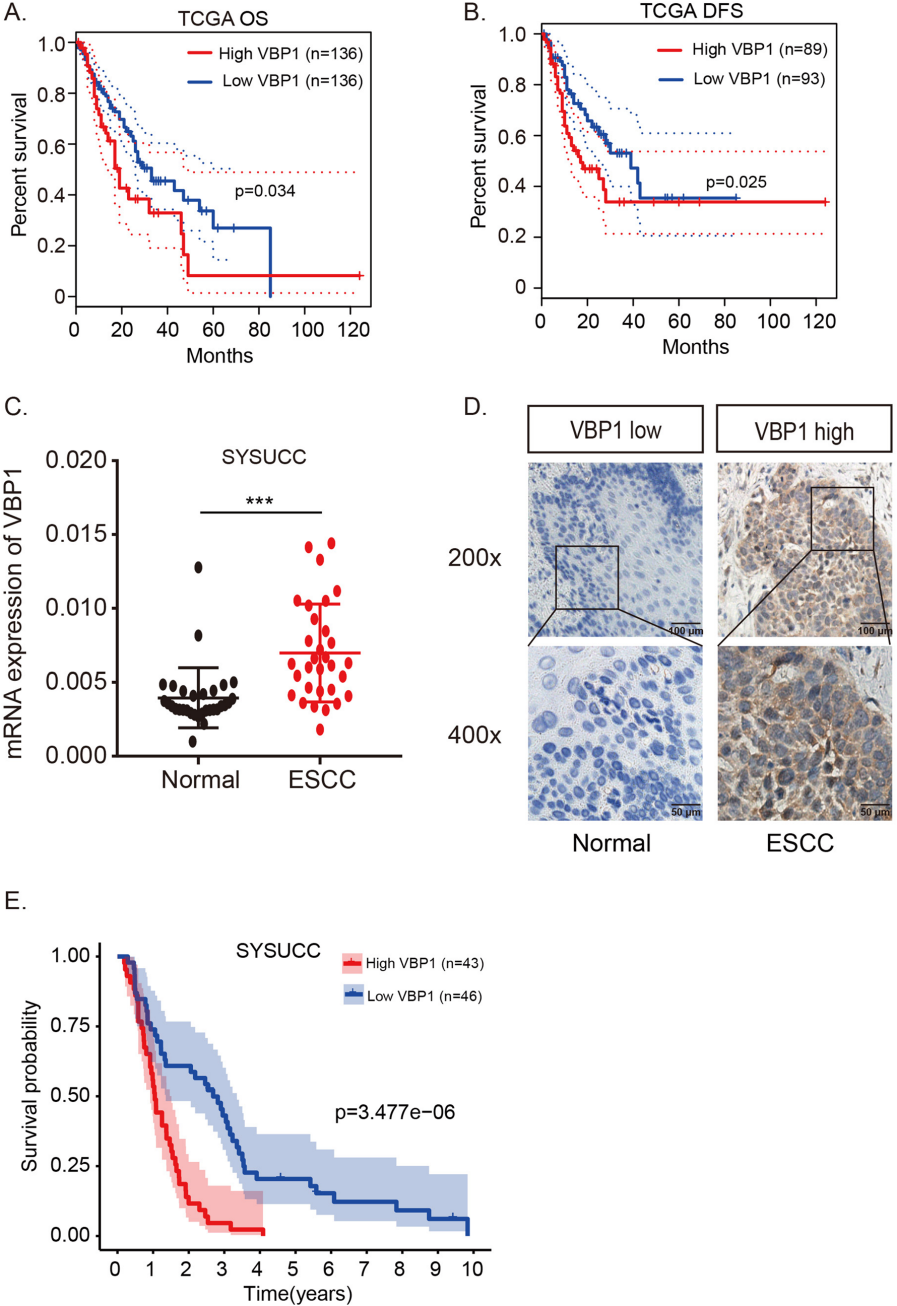
Evaluation of the expression and prognostic value of VBP1 in ESCC. **A** Kaplan–Meier plots of VBP1 expression level of OS in TCGA ESCC patients (log rank test, p = 3.4e-02). **B** Kaplan–Meier plots of VBP1 expression level of DFS in TCGA ESCC patients (log rank test, p = 2.5e-02). **C** The mRNA expression level of VBP1 identified by qRT-PCR in ESCC and normal tissues (paired t-Test, p = 4.0e-03). **D** The protein expression level identified by immunohistochemistry of VBP1 in ESCC and normal tissues. **E** Kaplan–Meier plot of VBP1 expression level scored by IHC in SYSUCC ESCC cohort (log rank test, p = 3.477e-06). *OS* Overall Survival, *DFS* Disease Free Survival, *qRT-PCR* Real‑Time quantitative polymerase chain reaction, *IHC* immunohistochemistry, *SYSUCC* Sun Yat-Sen University Cancer Center

We cross-referenced the four genes (VBP1, BGN, CDKN1A, and PPFIA1) in the GEPIA database to validate the prognostic significance of the hypoxia-related signature. Among them, only VBP1's expression exhibited a correlation with both overall survival and disease-free survival in ESCC (Fig. 4A, 4B, Supplementary Fig. 1). And there is no clinical baseline bias was found in the database (Supplementary Table 4, 5). As such, we pinpointed VBP1 as a critical gene and delved deeper into its role in ESCC.

To understand the expression and clinical implications of VBP1 in ESCC, we evaluated VBP1 expression and its prognostic relevance in the SYSUCC cohort. Our findings indicated an elevated expression of VBP1 in ESCC compared to normal esophageal tissues at both mRNA and protein levels (Fig. 4C, 4D). Based on the IHC score and survival data, ESCC patients exhibiting high VBP1 expression encountered more adverse outcomes compared to those with lower VBP1 expression (P < 0.001, Supplementary Fig. 2, Fig. 4E). This underscores the premise that VBP1 upregulation in ESCC tumors aligns with a poor prognosis.

**Fig. 5:**
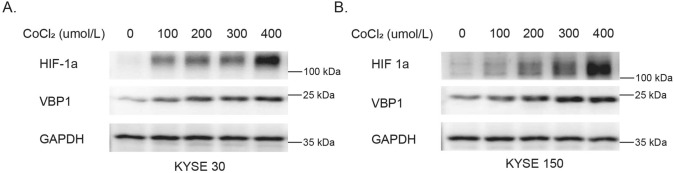
The protein expression of HIF-1α and VBP1 when treated with different concentrations of Cocl_2_ (0–400 umol/L). **A** The protein expression of HIF-1α and VBP1 is increasing in KYSE30 cells.** B** The protein expression of HIF-1α and VBP1 is increasing in KYSE150 cells

To establish the relationship between VBP1 and hypoxia, we simulated a hypoxic environment using CoCl_2_. This revealed that VBP1's protein level ascended concurrently with increasing concentrations of CoCl_2_ and the protein level of HIF1-ɑ(Fig. 5). Therefore, VBP1 was reaffirmed as a hypoxia-associated gene, potentially pivotal to the progression of ESCC.

### VBP1 accelerates ESCC proliferation both in vitro and in vivo

**Fig. 6:**
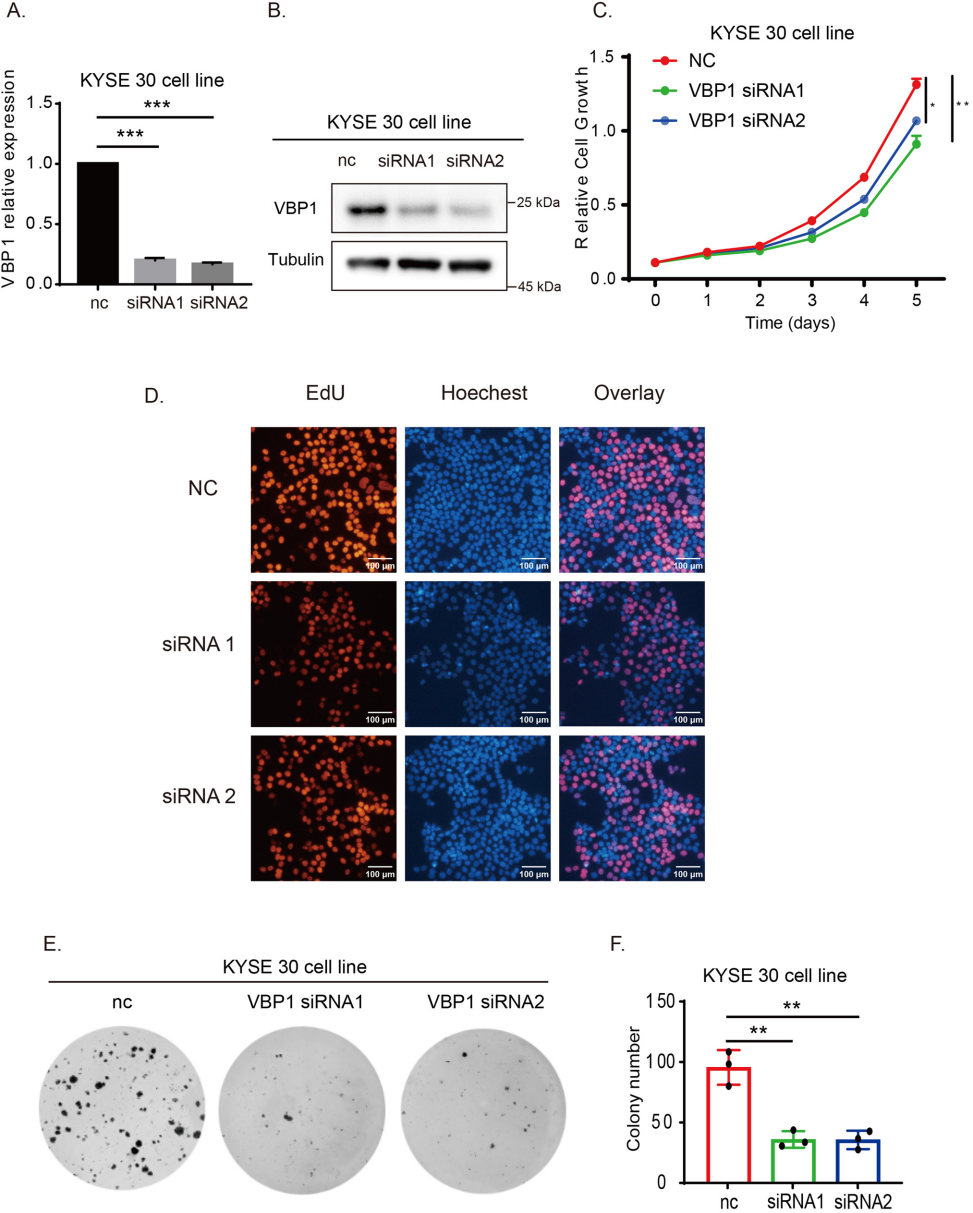
VBP1 promotes ESCC proliferation in KYSE30 cells. **A** The mRNA expression of VBP1 in NC and VBP1-siRNA group. **B** The protein expression of VBP1 in NC and VBP1-siRNA group. **C** CCK-8 assay showed that the proliferation is decreased in VBP1-siRNA group. **D** Edu assay showed that the proliferation is decreased in VBP1-siRNA group. **E** Plate clone formation assay showed that the proliferation is decreased in VBP1-siRNA group.** F** Plate clone formation assay was performed with three biological replicates, and the colony numbers of KYSE30 cells were counted and analyzed. *NC* normal control

**Fig. 7:**
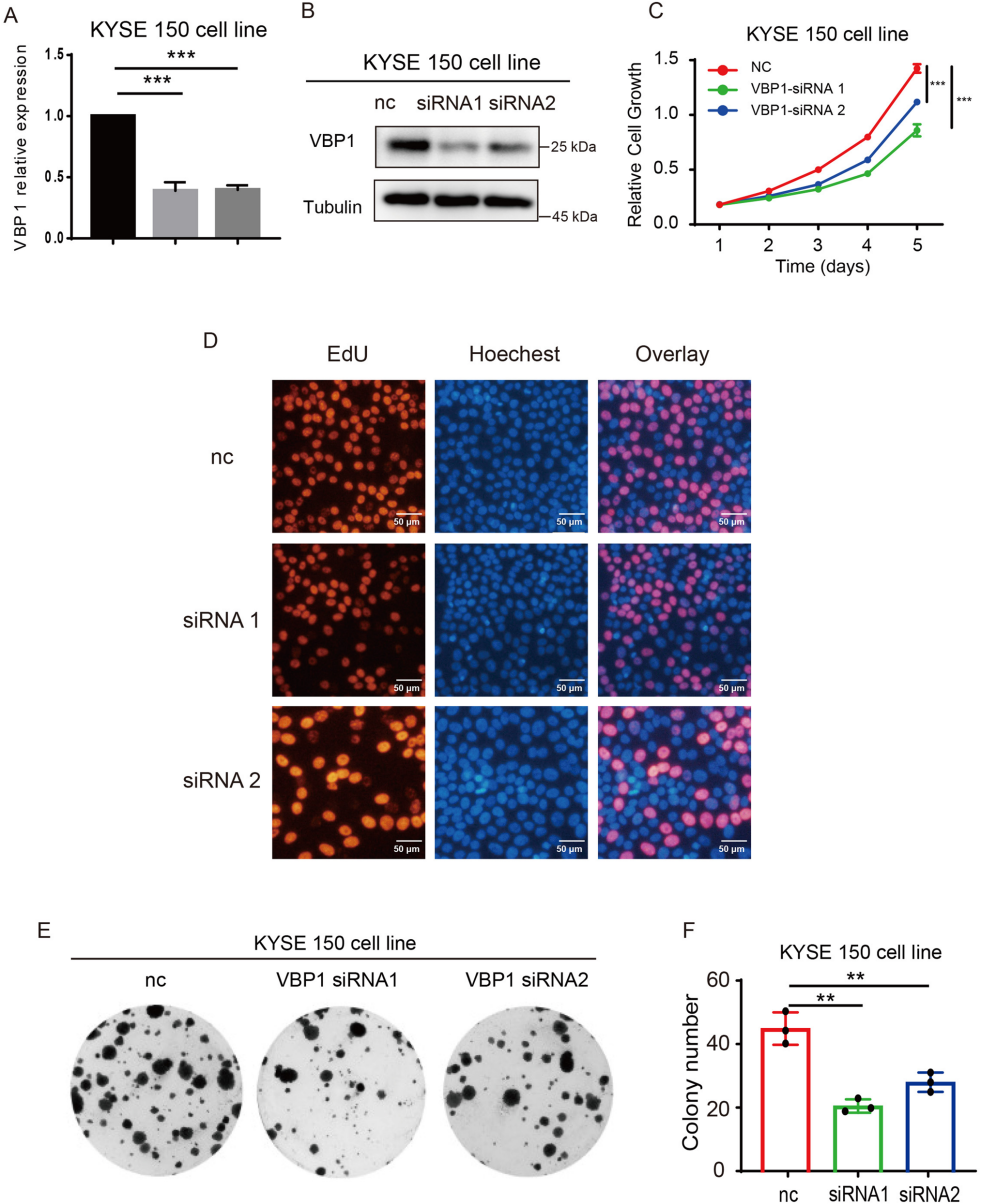
VBP1 promotes ESCC proliferation in KYSE150 cells. **A** The mRNA expression of VBP1 in NC and VBP1-siRNA group. **B** The protein expression of VBP1 in NC and VBP1-siRNA group. **C** CCK-8 assay showed that the proliferation is decreased in VBP1-siRNA group. **D** Edu assay showed that the proliferation is decreased in VBP1-siRNA group. **E** Plate clone formation assay showed that the proliferation is decreased in VBP1-siRNA group.** F** Plate clone formation assay was performed with three biological replicates, and the colony numbers of KYSE150 cells were counted and analyzed. *NC* normal control

**Fig. 8:**
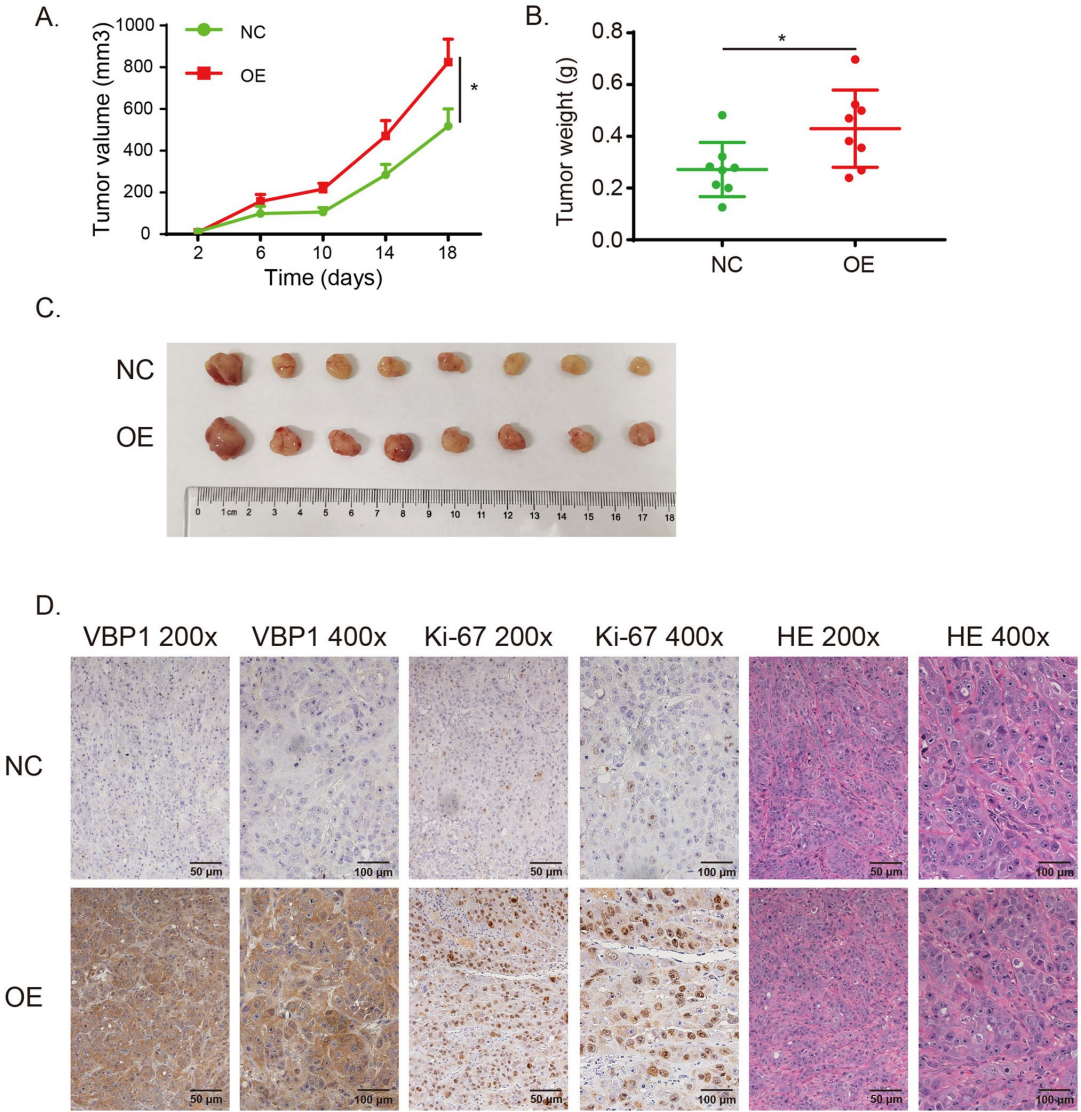
VBP1 promotes ESCC proliferation in nude mouse subcutaneous xenograft tumor. **A** The tumor volume is increased in VBP1-OE group. **B** The tumor weight is increased in VBP1-OE group. **C** The appearance of nude mouse subcutaneous xenograft tumor in VBP1-NC (n = 8) and OE (n = 8) groups. **D** The immunohistochemistry of VBP1 and Ki-67 in VBP1-NC and OE groups. *OE* overexpression; *n* number

To understand the role of VBP1 in vitro, KYSE30 cells were transfected with VBP1 siRNA (Fig. 6A, 6B). Proliferation assays such as CCK-8 (Fig. 6C), EdU (Fig. 6D), and colony formation (Fig. 6E) revealed that VBP1 interference curbed the proliferation of KYSE30 cells. The same observation was made with KYSE150 cells (Fig. 7). After establishing the influence of VBP1 on ESCC proliferation in vitro, we investigated its effect in vivo. We used KYSE30-NC and KYSE30-VBP1-OE cells to create a nude mouse subcutaneous xenograft model. Our findings indicated that the tumor growth rate and weight were notably higher in the VBP1-OE group than the NC group (Fig. 8A, 8B, 8C). Furthermore, immunohistochemical analysis of tumor tissues confirmed the elevated expression of VBP1 and Ki-67 in the VBP1 OE group (Fig. 8D).

## Discussion

ESCC is a major global contributor to cancer-related deaths due to its high recurrence and metastatic rates [1]. Its development is a multifaceted process influenced by numerous factors [19]. Notably, hypoxia stands out as a distinct feature of ESCC and other solid malignant tumors [20] [21]. Typically, solid tumor hypoxic regions display oxygen gradients, plunging from a normal 9% to less than 2% oxygen levels [5]. This hypoxic state induces a profound shift in the phenotype of cancer cells, changing their metabolism and replicative abilities [22]. One significant consequence of hypoxia is the increase of reactive oxygen species (ROS) in the electron transport chain [22]. This surge in ROS, coupled with disrupted redox equilibrium, can precipitate DNA mutations and genomic instability [20]. However, the precise roles of hypoxia and ROS in ESCC's evolution and progression remain to be elucidated.

Our study unveils a hypoxia-related signature (comprising VBP1, BGN, CDKN1A, and PPFIA1) that bears a strong correlation with ESCC. This signature acts as an independent predictor, offering high accuracy in prognosticating ESCC outcomes. Importantly, the genes within this signature play roles in DNA replication and homologous recombination, known mechanisms that drive tumor proliferation and poor ESCC prognosis. To the best of our knowledge, this is the first report of a hypoxia-related prognostic signature for ESCC.

VBP1, or VHL binding protein 1, functions as a chaperone protein for pVHL, facilitating protein transport from perinuclear granules to the nucleus [11]. While VBP1's involvement in colon and renal cell carcinoma progression is documented, its role in ESCC is less explored [23] [24]. Previous research tagged VBP1 as a positive regulatory protein for pVHL, which stabilizes pVHL by inhibiting its ubiquitination [23]. J. K. Ahn reported that VBP1 elevates pVHL-induced HIF-1α ubiquitination, destabilizing HIF-1α and potentially inhibiting tumor metastasis [25]. In our findings, hypoxic conditions in ESCC induce VBP1 expression. VBP1 is correlated with poor ESCC outcomes and is seen to foster tumor progression both in vitro and in vivo.

The dual nature of hypoxia in the tumor microenvironment warrants attention. While hypoxia can bolster tumor development and progression, it, alongside ROS, offers therapeutic avenues [26]. For instance, redox therapies, utilizing redox-active drugs or inhibitors, have shown promise against tumors resistant to multiple drugs [27]. In our context, VBP1 inhibition could halt tumor growth, signposting a potential therapeutic target. Delving deeper into redox biology and ROS manipulation in tumors may yield potent therapies against carcinomas.

## Conclusion

In our study, we developed a prognostic model rooted in four hypoxia-related genes that demonstrated robust predictive capabilities for ESCC. Furthermore, we pinpointed VBP1 as a pivotal gene within this hypoxia-related signature, acting as an oncogenic driver for ESCC progression.

### Supplementary Information

Below is the link to the electronic supplementary material.

Supplementary file1 (DOCX 2237 KB)

## Data Availability

Data will be made available on request.

## Declarations

### Registry and the registration no. of the study/trial

N/A.

### Reaeach involve human and animal

The animal study had approval from the Animal Care and Use Committee of Sun Yat-sen University Cancer Center (2021-000123). The guideline we followed for using animals is the ethical review of laboratory animal welfare (People’s Republic of China National Standard GB/T 35892-2018), which including the principles of Replacement, Reduction and Refinement.
