# Supplementary material for: VBP1 promotes tumor proliferation as a part of the hypoxia-related signature in esophageal squamous cell carcinoma
Source: Hum Cell. 2024 May 3;37(4):1141–55. doi: 10.1007/s13577-024-01068-9 (PMC11194215; doi:10.1007/s13577-024-01068-9)
Supplement: Supplementary file 1 — Supplementary file1 (DOCX 2237 KB) [file 13577_2024_1068_MOESM1_ESM.docx]

**Supplementary Figure 1:**

**Construction of the hypoxia‑related prognosis signature in ESCC**.


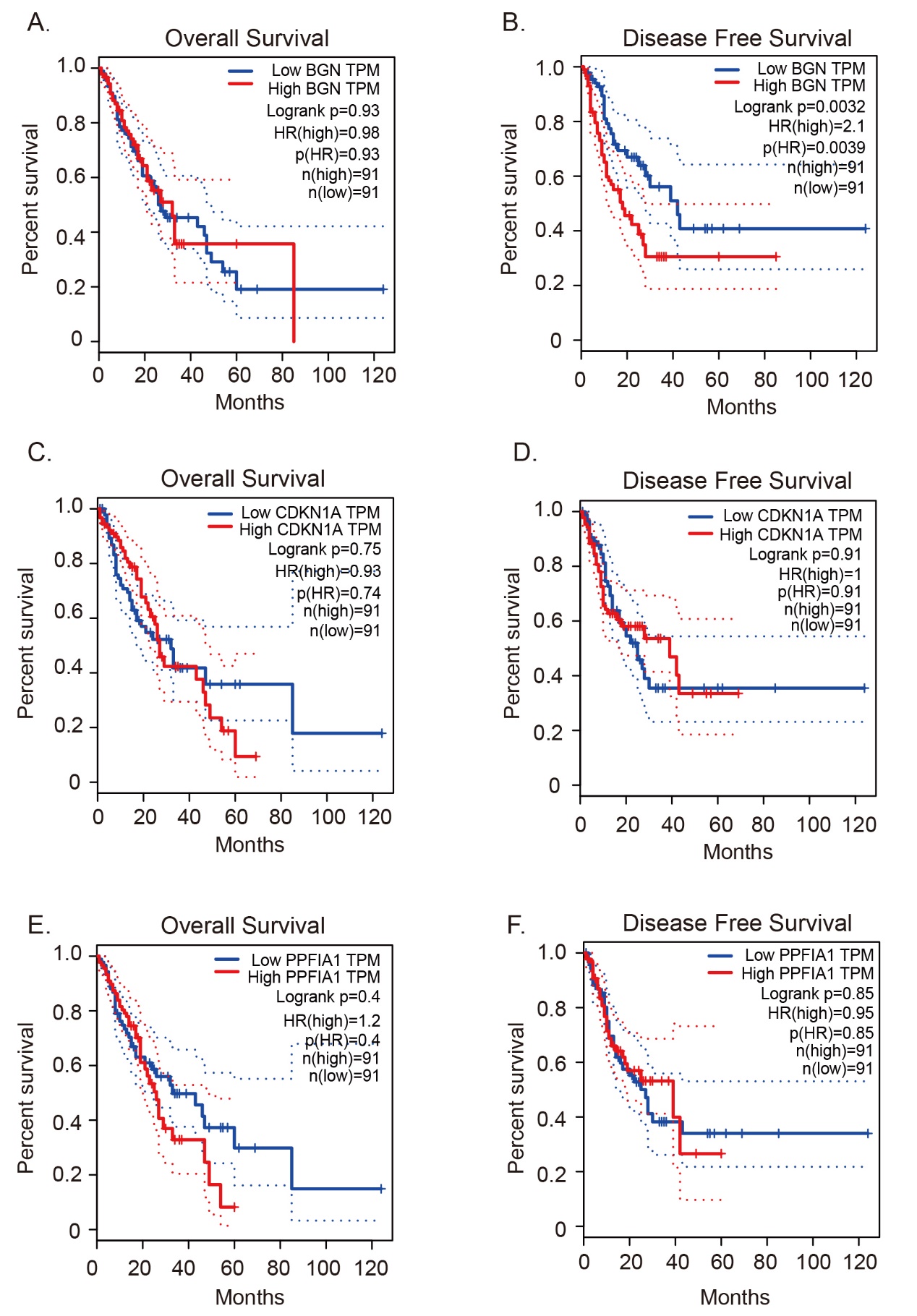


**Supplementary Figure 2:**

**Evaluation of the hypoxia‑related prognosis signature in ESCC.**


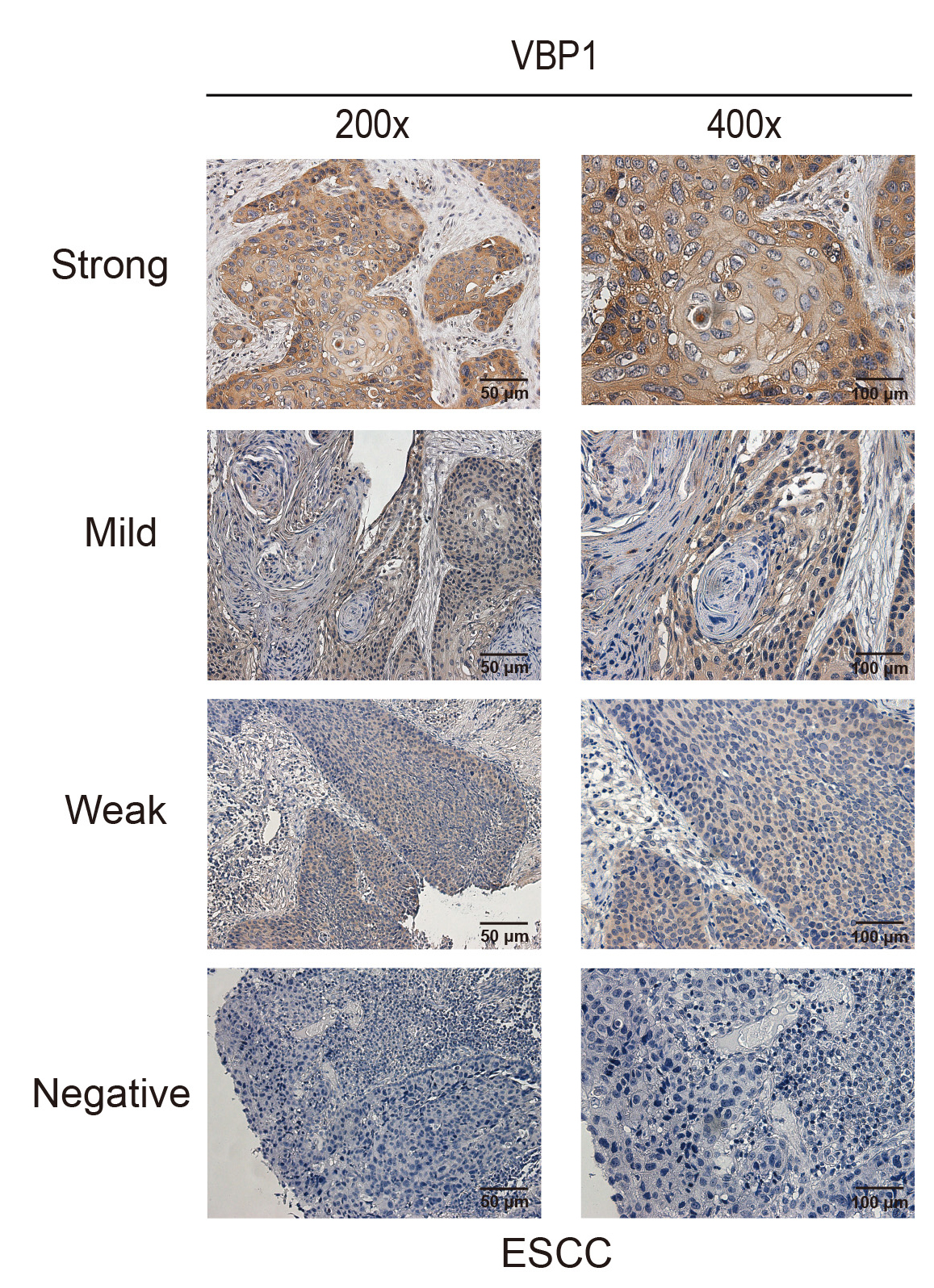


**Supplementary Table 1:**

**Antibodies details for IHC and WB.**

| **Primary antibody** | **Catalog number, Company** | **IHC** | | **WB dilution** |
| --- | --- | --- | --- | --- |
|  |  | ***Antibody dilution*** | ***Antigen retrieval buffer*** |  |
|  |  |  | TE buffer, pH 9.0  -  TE buffer, pH 9.0  -  - |  |
| VBP1 | 14345-1-AP, Proteintech, China | 1:500 |  | 1:1000 |
| HIF-1α | 20960-1-AP, Proteintech, China | - |  | 1:1000 |
| Ki-67 | 27309-1-AP, Proteintech, China | 1:2000 |  | - |
| GAPDH  Beta Tubulin | 10494-1-AP, Proteintech, China  10068-1-AP  Proteintech, China | -  - |  | 1:5000  1:5000 |

IHC, immunohistochemistry; WB, Western blot; TE, Tris-EDTA;

**Supplementary Table 2:**

**RT-qPCR primers sequences details.**

| **Technique** | **Gene** | **Forward sequence (5’-3’)** | **Reverse sequence (5’-3’)** |
| --- | --- | --- | --- |
| RT-qPCR | VBP1 | AGTCCACCAACTCAATGGAGA | CAAGCATTACATTAGCCCCCAA |
|  | ACTB | CACCATTGGCAATGAGCGGTTC | AGGTCTTTGCGGATGTCCACGT |
|  |  |  |  |

**Supplementary Table 3:**

**Small interfering RNA (si-RNA) sequences details.**

| **Technique** | **Gene** | **Sense (5’-3’)** | **Anti-sense (5’-3’)** |
| --- | --- | --- | --- |
| si-RNA knockdown | VBP1 siRNA1 | GGAAAUGGUAAUUUGCCUUTT | AAGGCAAAUUACCAUUUCCTT |
|  | VBP1 siRNA2 | CUGUUGCUAUUGUCUUCUAUA | UAGAAGACAAUAGCAACAGAG |
|  |  |  |  |
